# *K*-mer-based machine learning method to classify LTR-retrotransposons in plant genomes

**DOI:** 10.7717/peerj.11456

**Published:** 2021-05-19

**Authors:** Simon Orozco-Arias, Mariana S. Candamil-Cortés, Paula A. Jaimes, Johan S. Piña, Reinel Tabares-Soto, Romain Guyot, Gustavo Isaza

**Affiliations:** 1 Department of Computer Science, Universidad Autónoma de Manizales, Manizales, Caldas, Colombia; 2 Department of Systems and Informatics, Universidad de Caldas, Manizales, Caldas, Colombia; 3 Department of Electronics and Automation, Universidad Autónoma de Manizales, Manizales, Caldas, Colombia; 4 Institut de Recherche pour le Développement, CIRAD, Univ. Montpellier, Montpellier, France

**Keywords:** Transposable elements, LTR retrotransposons, Plant genomes, Machine learning, Classification, Free-alignment approach, *k*-mer based method

## Abstract

Every day more plant genomes are available in public databases and additional massive sequencing projects (i.e., that aim to sequence thousands of individuals) are formulated and released. Nevertheless, there are not enough automatic tools to analyze this large amount of genomic information. LTR retrotransposons are the most frequent repetitive sequences in plant genomes; however, their detection and classification are commonly performed using semi-automatic and time-consuming programs. Despite the availability of several bioinformatic tools that follow different approaches to detect and classify them, none of these tools can individually obtain accurate results. Here, we used Machine Learning algorithms based on *k*-mer counts to classify LTR retrotransposons from other genomic sequences and into lineages/families with an F1-Score of 95%, contributing to develop a free-alignment and automatic method to analyze these sequences.

## Introduction

The availability of large-scale biological data is changing the way researchers must analyze and find solutions to problems in almost every area of biological sciences. Machine Learning (ML) algorithms can use this data to automatically learn the parameters needed to fit a model to a specific problem (Shastry & Sanjay, 2020) in order to predict known labels. This process is called supervised learning (Zou et al., 2018). Bioinformatics, which is an intersection between computer sciences, biological sciences, and mathematics (Orozco-Arias et al., 2017), plays a central role in storing, analyzing, categorizing, and labeling the huge flow of information generated, for example, by next-generation sequencing (NGS) platforms. Advances in these sequencing technologies have provided a new paradigm in the field of post-genomics (Rigal & Mathieu, 2011; Chen et al., 2014; Rishishwar et al., 2017), which focuses on how to store, process, and analyze these data streams in acceptable time frames (Rigal & Mathieu, 2011; Chen et al., 2014; Rishishwar et al., 2017).

By automatizing tasks that were done manually, ML is impacting many areas of bioinformatics (Li et al., 2020), such as genomics (Libbrecht & Noble, 2015; Zou et al., 2018; Eraslan et al., 2019), systems biology (Larrañaga et al., 2006), and, specifically, the annotation of transposable elements (TEs) (Orozco-Arias et al., 2019). This last task is a current challenge in genomics (Ou, Chen & Jiang, 2018; Orozco-Arias et al., 2020). There is a growing interest in these repeated sequences due to their key functional and evolutionary roles on eukaryote genomes (Hesam & Ali, 2010; Orozco-Arias, Isaza & Guyot, 2019). Nevertheless, the detection and classification of these sequences remain complex because of their highly repetitive nature, diversity, polymorphism, species specificity, among other factors (Ou, Chen & Jiang, 2018; Mustafin & Khusnutdinova, 2018).

Although there is an open debate on how to classify TEs, the most classification approach is based on their lifecycle (i.e., how they move inside the genome). TEs are classified hierarchically (Orozco-Arias et al., 2019); first, they are divided into two main classes (e.g., Class I or retrotransposons and Class II or DNA transposons (Wicker et al., 2007)) and further divided into orders according to the similarity of their coding domains (Chaparro et al., 2015; Orozco-Arias, Isaza & Guyot, 2019; Neumann et al., 2019). In plants, the most frequent elements are, by far, long terminal repeat (LTR) retrotransposons (LTR-RTs, an order from Class I) (Gao et al., 2012; Grandbastien, 2015a), which account for 80% of the genome size of species such as wheat, barley, or the rubber tree (Rahman et al., 2013).

Indeed, bioinformatic approaches have developed many tools to detect and classify transposable elements, including multiple approaches that group TEs based mainly on their structure, homology, redundancy, or conservation across genomes (Rawal & Ramaswamy, 2011; Jiang & Ramachandran, 2013; Loureiro et al., 2013; Schietgat et al., 2018). Nevertheless, the complexity of these elements does not consistently allow for accurate, reliable, and reproducible results across programs for all types of TEs (Arkhipova, 2017). In recent years, several machine learning-based methods have been proposed and evaluated, which take advantage of the thousands of sequences available in several datasets, such as Repbase (Jurka et al., 2005), RepetDB (Cornut et al., 2019), PGSB (Spannagl et al., 2016a), and InpactorDB (Orozco-Arias et al., 2021).

Several studies have, therefore, proposed the use of ML for TE analysis (reviewed in (Orozco-Arias et al., 2019)) to differentiate between LTR-RTs and SINEs (Ashlock & Datta, 2012) or autonomous and non-autonomous LTR-RTs in the *Coffea canephora* genome (Arango-López et al., 2017), and to improve the accuracy and performance of the classification (Loureiro et al., 2013; Nakano et al., 2017). Recently, a random forest algorithm was used to broadly classify LTR-RTs into superfamilies (Schietgat et al., 2018), while additional pre-processing techniques and coding schemes allow their deep classification (Orozco-Arias et al., 2020). Moreover, several deep neural network (DNN) architectures that perform TE classification have been published so far. For instance, Nakano et al. implemented a fully connected neural network (FNN) (Nakano et al., 2018), da Cruz et al. used a convolutional neural network (CNN) with a 2D representation of the sequences (da Cruz et al., 2019), and Yan et al. used a CNN in 1D to classify TEs into superfamilies (da Cruz et al., 2020; Yan, Bombarely & Li, 2020).

Despite these efforts, none consider the need to both detect and classify elements at the same time, or specifically aim to classify LTR-RTs to the lineage/family level. In this paper, we evaluate and demonstrate the application of ML algorithms in the binary classification between LTR-RTs and other genomic features, multi-class classification into lineages/families or both (in a single process), as well as the importance of the features used, in order to design a free-alignment method for the annotation of LTR-RT in plant genomes based on *k*-mer frequencies.

## Materials & Methods

### Dataset composition

We used InpactorDB ((Orozco-Arias et al., 2021), DOI 10.5281/zenodo.4386316 or 10.23708/QCMOUA), which comprises 67,241 LTR retrotransposon sequences, deeply classified into lineages/families, from 195 plant species. This dataset initially contained sequences from Repbase, RepetDB, and PGSB, which were processed using several filters to remove low quality elements (i.e., elements with nested sequences) (Orozco-Arias et al., 2021). It also contained LTR-RTs predicted by LTR_STRUC (McCarthy & McDonald, 2003) and EDTA (Ou et al., 2019). As negative instances, we created a dataset composed of annotated genomic features other than LTR_RTs, such as coding sequences (CDS), different types of RNA (e.g., mRNA, tRNA, non-coding RNA, among others), and other types of transposable elements that do not belong to LTR-RTs (e.g., TIR, Helitron, PLEs, DIRs, LINEs, and SINEs) from the same plant species contained in InpactorDB. These additional TE sequences were available in databases such as PGSB PlantsDB (Spannagl et al., 2016b), Repbase (v. 20.05, 2017) (Bao, Kojima & Kohany, 2015), RepetDB (Amselem et al., 2019), Ensembl Plants (Bolser et al., 2017), and JGI (Joint Genome Institute) (Nordberg et al., 2014) (Supplemental Material 1). This dataset is available in DOI 10.5281/zenodo.4543904.

For the binary classification task, we randomly selected 10,000 LTR retrotransposon sequences (taken as positive instances) and 10,000 genomic feature sequences for the negative instances. For the classification task into lineages/families, we used only InpactorDB data, while, for the binary plus multi-class classification problem (unified at a single ML process), we filtered the negative instances to retain only sequences longer than 6 Kb. We did this filter in order to reduce the number of sequences from more than 3 million to 34,830 and because the average length of Copia elements in InpactorDB is 5,957.48. In contrast the average length of Gypsy elements is 10,760.57.

As features, we selected *k*-mer frequencies with 1<=k<=6, as recommended in Orozco-Arias et al. (2020), calculating all possible *k*-mers, and later counting the number of occurrences of them in each sequence. We calculated them for lineage-level classification and the binary plus multi-class classification task. For binary classification, we used the same coding schemes as implemented in Orozco-Arias et al. (2020), such as DAX (Yu et al., 2015), EIIP (Nair & Sreenadhan, 2006), Complementary (Akhtar, Epps & Ambikairajah, 2008), Enthalpy (Kauer & Blöcker, 2003), and Galois (4) (Rosen, 2006). Additionally, two techniques were applied to automatically extract features from the sequences: (i) *k*-mer frequencies were obtained for each element and (ii) three physical-chemical (PC) properties were extracted, such as average hydrogen bonding energy per base pair (bp), stacking energy (per bp), and solvation energy (per bp), which were calculated by taking the first di-nucleotide and moving in a sliding window of one base at a time (Jaiswal & Krishnamachari, 2019). Moreover, we pre-processed the data by scaling, following the strategy implemented in Tabares-soto et al. (2020), and performed a dimensional reduction through a principal component analysis (PCA) (Wold, Esbensen & Geladi, 1987) with a cumulative variance of 96% and tolerance of 1e−4.

For the binary classification task, we divided the dataset into a training set (80% of the data) a validation set (10%), and a test set (10%). For multi-class classification into lineages, we used the same partition and additionally, we used k-cross-validation (Komer, Bergstra & Eliasmith, 2014) with k = 9 after tuning hyper-parameters in order to test the generalization property of each model.

### Machine learning algorithms used

For binary classification between positive (LTR retrotransposons) and negative (other genomic features) instances, we used the same algorithms described in Orozco-Arias et al. (2020); thus, we used Linear Support Vector Classifier (SVC), Logistic Regression (LR), Linear Discriminant Analysis (LDA), K-Nearest Neighbors (KNN), Naive Bayesian Classifier (NB), Multi-Layer Perceptron (MLP), Decision Trees (DT), and Random Forest (RF) and selected the larger F1-Score for different values of a hyper-parameter (as in Orozco-Arias et al. (2020)).

For classification, we used the supervised models that showed the best performance in Orozco-Arias et al. (2020), such as KNN, LR, SVC, and LDA, but we applied hyper-parameter tuning (Table 1) using GridSearchCV from Scikit-learn (Pedregosa et al., 2011), using only sequences from InpactorDB (we did not include negative instances due to the high memory required). We used the F1-Score as a performance metric in all executions since it is not affected much by unbalanced datasets such as LTR-RTs (Orozco-Arias et al., 2020).

**Table 1:** Machine Learning models and hyper-parameters tuned.

Classifier	Parameter	Range
**KNN**	neighbors	2,20,39,57,76,94,113,131,150
	weights	uniform, distance
	metric	euclidean, manhattan, chebyshev, minkowski, wminkowski, seuclidean, mahalanobis
	algorithm	auto, ball_tree, kd_tree, brute
**Linear SVC**	C	1 × 10^i^ with i = −4, to 5
	penalty	l1, l2
	loss	hinge, squared_hinge
	tol	10^−1^, 10^−2^, 10^−4^, 10^−8^
**LR**	C	1 × 10^i^ with i = −4, to 5
	tol	1 × 10^i^ with i = −4, to 5
	max_iter	1 × 10^i^ with i = 0, to 6
	penalty	l1, l2, elasticet, none
	solver	saga, liblinear, newton-cg, lbfgs, sag, saga
**LDA**	shrinkage	1, 0.1, 0.5, 0.001, 0.0001, 0.00001
	solver	svd, lsqr, eigen
	tol	10^−1^, 10^−2^, 10^−4^, 10^−8^

**Figure 1:**
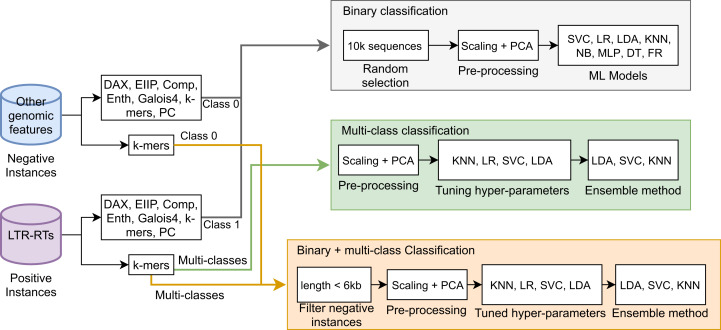
Schematic representation of the different approaches used in this study in the binary and multiclass classification tasks in LTR retrotransposons through Machine Learning.

Next, a Stacking Classifier was implemented as an ensemble algorithm, which is a combination of multiple ML models for creating a more complex model (Zhang & Ma, 2012; Müller & Guido, 2016). The stacking classifier comprised LDA, Linear SVC, and KNN algorithms and used Random Forest as a meta-classifier. Similarly, for binary plus multi-class classification, we used a Stacking Classifier with KNN, LDA, and LR algorithms and Random Forest as a meta-classifier. Figure 1 summarizes the three approaches used in this study. The implementation in Python 3 of all algorithms used in this study is available in the Supplemental Material 2 or at https://github.com/simonorozcoarias/MachineLearningInTEs/blob/master/Scripts/binary_plus_multi_clasification.py.

### Feature selection

We used the Gradient Boosting algorithm (Friedman, 2002) implemented in Scikit-Learn to determine the importance of each feature using the complete dataset (InpactorDB plus negative instances). Gradient Boosting generates scores for each feature that represent how useful it was in the construction of the boosted decision trees. The more valuable the feature is for making key decisions, the higher its importance score (Hastie, Tibshirani & Friedman, 2009). Thus, we extracted features with an importance score greater than 60, 40, 30, 20, 10, which yielded 65, 289, 508, 1,034, and 2,397 features from 5,460 *k*-mers. We used the following hyper-parameters: boosting_type=’goss’, n_estimators=10000, and class_weight=’balance’. Finally, we extracted the selected features in order to create new reduced datasets that were used to train the same ensemble algorithm implemented in the binary plus multi-class classification task.

## Results

### Binary classification of LTR retrotransposons and other genomic features

**Figure 2:**
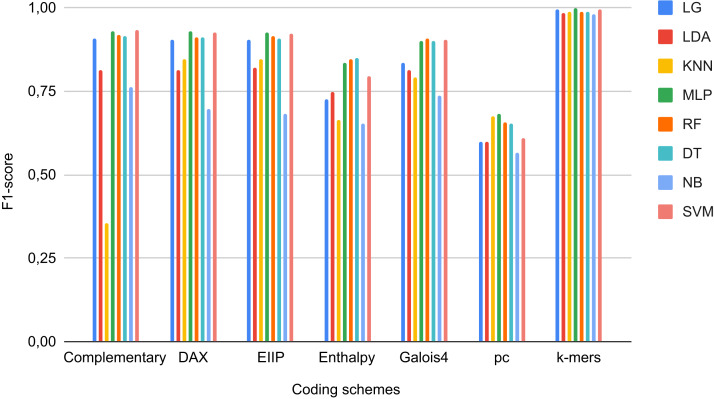
F1-Scores obtained by Machine Learning models using different coding schemes for the test set.

As negative instances, we obtained 2,713,028 coding sequences (CDS), 262,925 RNAs of different types (e.g., ncRNA, mRNA, miRNA, rRNA, snRNA, and tRNA), 37,077 TEs that did not correspond to LTR retrotransposons (i.e., TEs class II, LINEs, SINEs, DIRS, and PLEs), and 3566 quimeric sequences from Repbase, PGSB, and RepetDB (Table 2). These sequences, with the exception of TEs (outside LTR-RTs), were obtained from 47 plant species available in public databases, such as Ensembl Plants and JGI (Supplemental Material 1). We used sequences from InpactorDB as positive instances. Due to the high imbalance between the two instances (2,979,519 negative vs 67,241 positive), we randomly extracted 10,000 sequences from each class. Then, the DNA sequences were converted to numerical representations using the coding schemes and automated techniques described in Orozco-Arias et al. (2020). Finally, we applied data scaling and a dimensional reduction through PCA (Tabares-soto et al., 2020). Using this dataset, we trained ML algorithms and determined their performance in terms of the F1-Score of each coding scheme over each ML model (Fig. 2). For the binary classification task, we obtained F1-Scores up to 97.9% 96.3%, and 95.9% for MLP, SVC, and LR, respectively, in the test dataset using *k*-mer frequencies as features.

**Table 2:** Composition of the negative instances dataset.

TE type	Order	Superfamily	Number
**Class I (retrotransposons)**	Non-LTR	LINEs	767
	Non-LTR	SINEs	551
	PLEs	Penelope	297
	DIRS	DIRS	356
	DIRS	VIPER	1
	DIRS	Ngaro	2
	Unclassified	Unclassified	1,039
	**TOTAL**		**3,013**
**Class II (DNA transposons)**	TIR	Tc1-Mariner	2,326
	TIR	hAT	2,587
	TIR	Merlin	65
	TIR	Transib	119
	TIR	PiggyBac	19
	TIR	PIF – Harbinger	973
	TIR	MuDR	1,016
	TIR	CACTA	57
	TIR	En-Spm	1,265
	TIR	MITE	2,312
	Crypton	Crypton	231
	Helitron	Helitron	740
	Unclassified	Unclassified	22,354
	**TOTAL**		**34,064**
**Non-TEs**	RNA		262,925
	CDS		2,713,028
	Quimeric sequences		3,566
	**TOTAL**		**2,979,519**

### Multi-class classification of LTR retrotransposons into lineages/families

**Figure 3:**
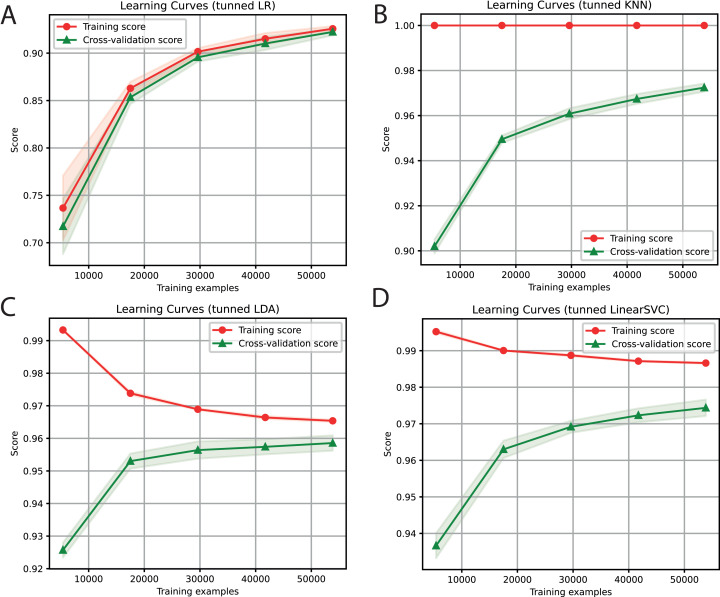
Learning curves of tuned ML algorithms: LR (A), KNN (B), LDA (C), and Linear SVC (D), to classify LTR retrotransposons up to the lineage level. The shadow on the lines indicates the standard variation.

For each of the selected models, a dictionary was created containing the hyper-parameters and the values to be iterated. After training each ML algorithm with GridsearchCV (Tabares-soto et al., 2020), we determined the parameters that generated the best performance, as shown in Table 3. After tuning the hyper-parameters, each model was retrained to determine its performance. We obtained F1-Scores of 91%, 97%, 96%, and 97% with LR, KNN, LDA, and SVC algorithms, respectively (Fig. 3).

**Table 3:** Tuned parameter values.

Classifier	Parameter	Value
**KNN**	neighbors	2
	weights	distance
	metric	euclidean
	algorithm	auto
**Linear SVC**	C	0.001
	penalty	l2
	loss	squared_hinge
	tol	0.1
**LR**	C	0.01
	tol	10
	max_iter	1000
	penalty	l2
	solver	sag
**LDA**	shrinkage	0.0001
	solver	lsqr
	tol	0.1

**Figure 4:**
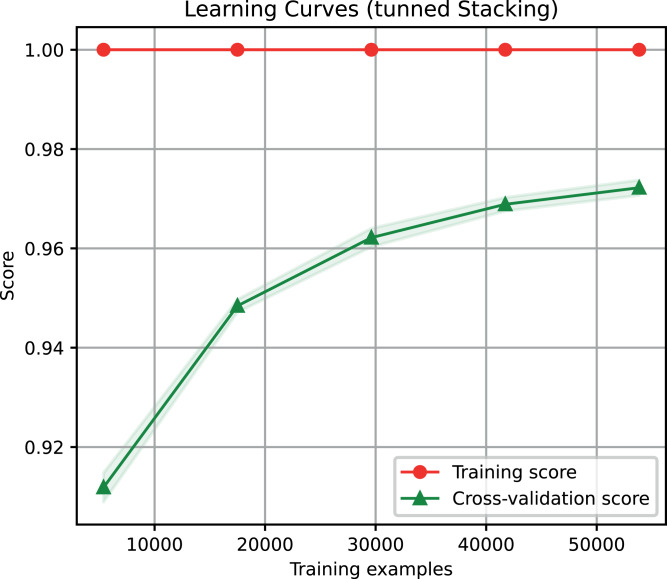
Learning curves of the Stacking classifier algorithm in the classification of LTR retrotransposons up to lineage/family level. The shadow on the line indicates the standard variation.

For the ensemble algorithm, the LR classifier was excluded since it showed the lowest performance (Fig. 3A). Therefore, the Stacking Classifier was implemented as an ensemble algorithm, composed of LDA, Linear SVC, and KNN algorithms, using Random Forest as meta-classifier. The performance of this ensemble model resulted in a 97% F1-Score, accuracy, recall, precision (Fig. 4), and 99% in area under ROC (receiver operating characteristic) curve (AUC) (Fig. S1) for the classification of LTR retrotransposons.

### Binary plus multi-class classification task

**Figure 5:**
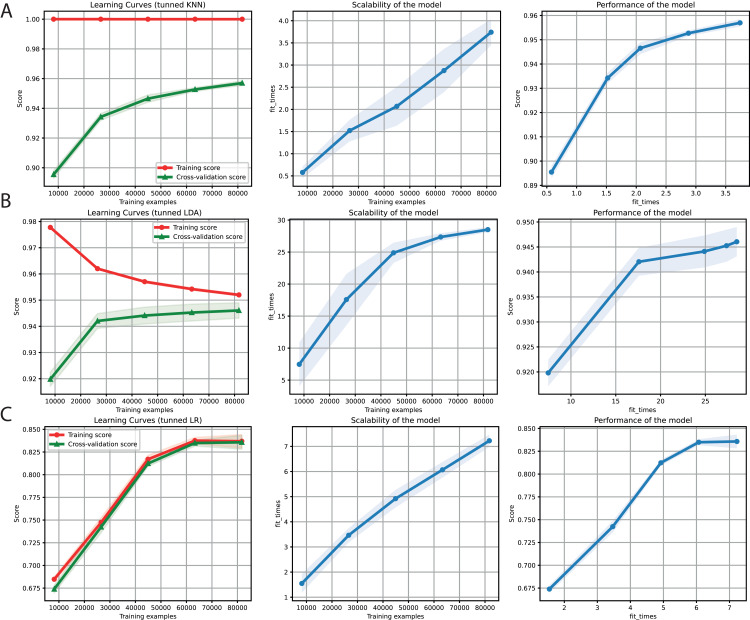
Learning curves for KNN (A), LDA (B), and LR (C) for the binary plus multiclass classification task. The shadow on the lines indicates the standard variation.

After obtaining promising results in both the binary and multi-class classification tasks, we proceeded to merge them into a single ML problem. Thus, we included the negative instances as another class but deleted the sequences with a length of less than 6 Kb (Table 4). Furthermore, only *k*-mers frequencies were used as features because of the high performance obtained for the two problems separately. We also used the hyper-parameter values tuned (Table 3) for KNN, LDA, and LR (Fig. 5). Finally, F1-Scores of 95%, 94%, and 84% were obtained using KNN, LDA, and LR, respectively.

**Table 4:** Dataset composition used in the binary plus multiclass classification task.

Class	Superfamily	Name	Number of classes
0	–	Other genomic features	34,823
1	Copia	ALE/Retrofit	12,031
3	Copia	Angela	1,458
4	Copia	Bianca	1,827
8	Copia	Ikeros	84
9	Copia	Ivana/Oryco	3,556
12	Copia	Tork/Tar	6,180
13	Copia	SIRE	3,130
		**Total Copia**	**28,266**
14	Gypsy	CRM	2,136
16	Gypsy	Galadriel	549
17	Gypsy	Reina	4,532
18	Gypsy	Tekay/DEL	10,396
19	Gypsy	Athila	3,499
20	Gypsy	TAT	17,927
		**Total Gypsy**	**39,039**

**Figure 6:**
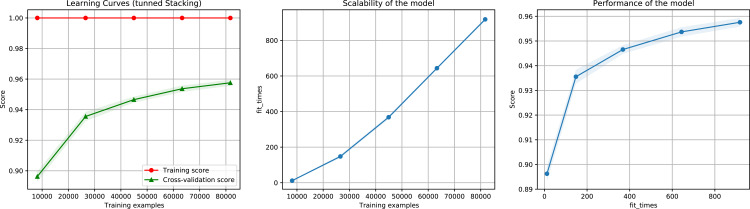
Learning curves for the Staking Classifier (ensemble method) for the binary plus multiclass classification task. The shadow on the lines indicates the standard variation.

**Figure 7:**
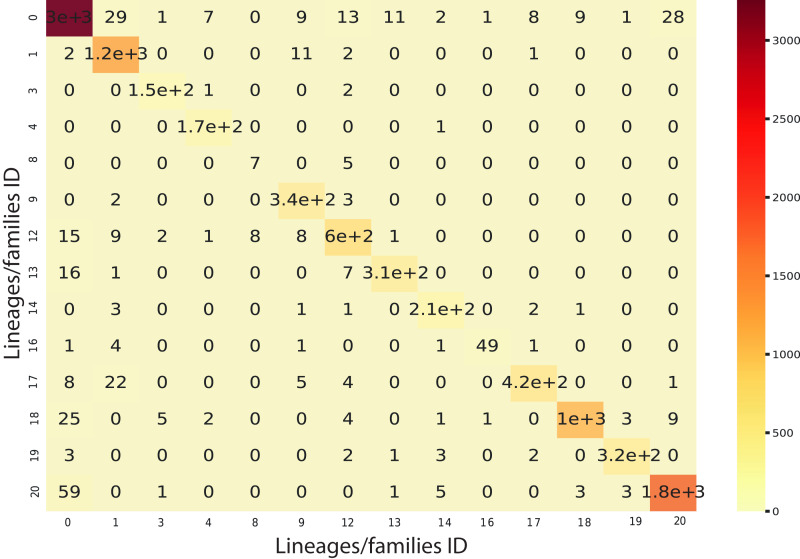
Confusion matrix for the Staking Classifier (ensemble method) used for the binary plus multiclass classification task. Each class (between 0 and 20) corresponds to the negative instances (for class 0) or to a linage/family otherwise (See Table 4).

We implemented an ensemble method (the same implemented for the classification task) using the three algorithms aforementioned and used RF as a meta-classifier. We obtained an F1-Score of 96% in k-cross validation with k = 9 (Fig. 6). This method also obtained 95% of precision and recall and 98% in AUC (Fig. S2). Furthermore, as shown in Fig. 7, the classes with the lowest F1-Scores are Class 8 (Ikeros, Copia) and 16 (Galadriel, Gypsy) since these classes have the lowest number of samples.

### Feature selection and evaluation

**Figure 8:**
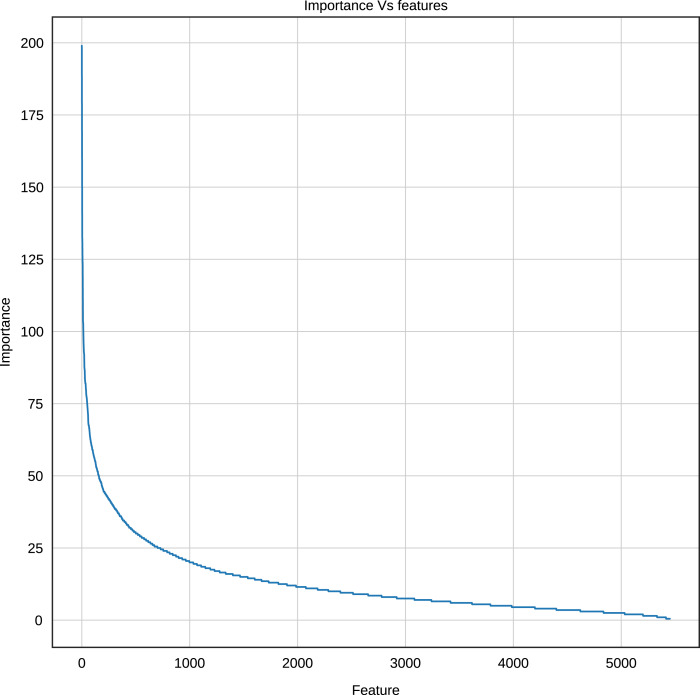
Importance score of the features (*k*-mer frequencies with 1<=k<=6).

Using the Gradient Boosting algorithm and the entire dataset (negative instances plus InpactorDB), we obtained the importance of each feature (*k*-mers frequencies). The number of features is relatively high (5,460). Since the computational cost to process them can be very high, the number of features must be reduced without reducing the performance of the ML algorithm. Figure 8 shows the importance of all features determined by Gradient Boosting.

**Figure 9:**
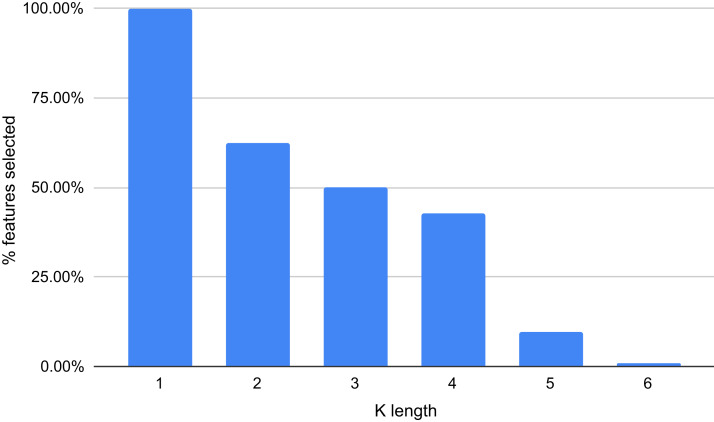
Percentage of features selected based on each value of k (between 1 and 6).

The results displayed in Fig. 8 demonstrate that some features are not relevant to the binary plus multi-class classification task. We extracted those with an importance score greater or equal to 40, thus, retaining only 289 features out of 5,460 (5.29%). The 10 most important features are: A, T, AAAAAA, ATAT, AGGGGG, CCCCC, TTTTTT, AGCT, GATC, GATGA with importance scores of 199, 179.5, 165, 140, 132.5, 132, 125.5, 124, 124, 114.5, respectively. Among the 289 selected features, we observed that increasing the length of k decreases the percentage of top selected features with greater importance (Fig. 9). In total, the 289 selected features were composed of 4, 10, 32, 109, 97, 37 of *k*-mers generated using k = 1,2,3,4,5 and 6, respectively (Supplemental Material 3).

**Figure 10:**
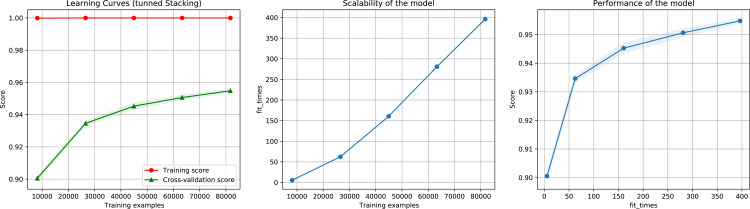
Learning curves for the Staking Classifier (ensemble method) for the binary plus multiclass classification task using only 289 selected features. The shadow in the lines indicates the standard variation.

Then, we executed the ensemble method again using the reduced dataset containing the most important features. We also test different importance score thresholds in order to keep different number of features. We used the same pre-processing technique and hyper-parameter values of the previous execution. The results show that reducing the number of features to 1.73% (from 5,460 to 95), 5.29% (289 features), 9.3% (508 features), 18.93% (1,034 features), and 43.9% (2,397 features), did not considerably decrease performance, as indicated by a 93.5%, 95.2%, 95.6%, 95.4%, 95.6% F1-Score (Fig. 10), accuracy, recall, and precision, using as importance score threshold 60, 40, 30, 20, and 10 respectively. We also noted that using 289 features we obtained an 97% in AUC (Fig. S3).

## Discussion

Transposable element detection and classification are time-consuming tasks for plant genome annotation projects and involve semi-automatic pipelines and curation by experts. These pipelines generally rely on sequence alignment approaches, which have a significant computational cost in the analysis of large genomes or TE compound genomes. The development of automatic algorithms for TE detection and annotation, without sequence alignment, is now required to process the huge amount of genomic information that is being generated.

The most frequent repeated sequences in plant genomes are LTR retrotransposons (Gao et al., 2012; Grandbastien, 2015b) Their transcriptional activities and mobilities can have a profound impact on the structure, composition, and size of genomes, among others (Orozco-Arias, Isaza & Guyot, 2019). To detect these elements in assembled genome sequences, the most frequent methods are based on their specific internal structure (e.g., the duplication of their LTR at both ends of the element) and domains, or via homology searches against reference libraries (Loureiro et al., 2013; Nicolas, Peterlongo & Tempel, 2016). Nevertheless, these strategies have strong limitations (Orozco-Arias, Isaza & Guyot, 2019). Structure-based methods will not accurately detect partial elements, variations, or non-autonomous elements such as solo-LTR, TR-GAG (Chaparro et al., 2015), or TRIM (Witte et al., 2001). Moreover, homology-based methods can induce low quality annotations if the reference library is incomplete or constructed with distant species of the species to be annotated (Orozco-Arias, Isaza & Guyot, 2019). These problems can be overcome using new methods, such as machine learning methods, that are not based on structure or homology (i.e., sequence alignment) (Nakano et al., 2018; Schietgat et al., 2018; Orozco-Arias et al., 2019; da Cruz et al., 2020). Although some studies have used ML to analyzed TEs, none of them have focused on the detection and lineage/family level classification of LTR retrotransposons. In previous studies (Orozco-Arias et al., 2020, 2021), we have shown that the classification of these elements can be very efficient with ML approaches but that coding schemes and feature extraction techniques can deeply influence the performance of such algorithms.

Here, we have shown the possibility of both binary and multi-class classification through ML with different approaches. First, by using a negative dataset (i.e., sequences of genomic features excluding LTR-RTs), we obtained an F1-Score up to 97.9% in the binary classification task. In the detailed lineage/family level classification task, we obtained a performance of 97% by using the same datasets as in Orozco-Arias et al. (2020) but including more elements from InpactorDB. Finally, we unified both tasks into a single process, obtaining a 96% F1-Score. These results were achieved using the *k*-mer frequencies of sequences. *K*-mers are frequently used in bioinformatics in the areas of quality control of generated sequences (Mapleson et al., 2017), metagenomics (Breitwieser, Baker & Salzberg, 2018), de novo assembly of genomes and transcriptomes (Zerbino & Birney, 2008; Simpson et al., 2009), genome size estimation (Sun et al., 2018), and de novo detection of transposable elements (Price, Jones & Pevzner, 2005). In addition to detection, *k*-mers have also demonstrated their usefulness in higher hierarchical classification approaches (at the superfamily level). Nakano and colleagues (Nakano et al., 2018) exploited the characteristics of *k*-mer frequencies (with k = 2,3,4) as features to train a deep neural network, and Yan, Bombarely & Li (2020) demonstrated interesting statistical differences using *k*-mer frequencies with k ranging from 3 to 7. Here, we found that the combination of certain *k*-mer frequencies with different values of k (from 1 to 6) added reliability to the binary plus multi-class classification task, requiring only 289 features (out of the initial 5,460 features) to obtain an F1-Score of 95%. We also demonstrated that, among all possible *k*-mers generated, larger k values reduce the number of features with high importance scores. The specific *k*-mer that contributes the most to the binary and multi-class classification characteristics must be analyzed in detail to understand its involvement in the composition and structure of the elements of each lineage. This work highlights the feasibility of designing and implementing ML-based tools that automate the complex process of annotating LTR retrotransposons in plant genomes. As future work, we propose the implementation of benchmarking to compare this method with well-established methods such as those based on homology and structure in various plant genomes.

## Conclusions

Massive sequencing projects require automatic tools to analyze large amounts of genomic information in a fast yet accurate, reliable, and reproducible manner. The binary classification of LTR-RTs and other genomic features and lineage-level classification of them in plant genomes can be performed using ML-based and ensemble methods, demonstrating good performance (up to 96% F1-Score). This task can be performed with only 289 *k*-mer frequencies, allowing low computational resources and time. These results can be used in the design and implementation of automatic and alignment-free tools to solve the issue of processing the increasing number of available plant genomes.

## Supplemental Information

10.7717/peerj.11456/supp-1 Supplemental Information 1 Plant species and URL from where they were downloaded for the creation of the negative instances.

10.7717/peerj.11456/supp-2 Supplemental Information 2 Features (*k*-mers) selected as contributing most to the binary plus multiclass classification task.

10.7717/peerj.11456/supp-3 Supplemental Information 3 Curve ROC for Stacking method in the multiclass classification problem. Each class (between 1 and 20) corresponds to a linage/family (See Table 4).

10.7717/peerj.11456/supp-4 Supplemental Information 4 Curve ROC for Stacking method in the binary plus multiclass classification problem. Each class (between 0 and 20) corresponds to the negative instances (for class 0) or to a linage/family otherwise (See Table 4).

10.7717/peerj.11456/supp-5 Supplemental Information 5 Curve ROC for Stacking method in the binary plus multiclass classification problem using the 289 features selected as most informative. Each class (between 0 and 20) corresponds to the negative instances (for class 0) or to a linage/family otherwise (See Table 4).

## Additional Information and Declarations

### Data Availability

The following information was supplied regarding data availability: The implementation in Python 3 of all algorithms is available at GitHub: https://github.com/simonorozcoarias/MachineLearningInTEs/blob/master/Scripts/binary_plus_multi_clasification.py. The positive instances dataset (LTR-Retrotransposons) is available at Zenodo: Orozco-Arias Simon, Jaimes Paula A., Candamil Mariana, Jiménez-Varón Cristian Felipe, Tabares-Soto Reinel, Isaza Gustavo, & Guyot Romain. (2021). InpactorDB: A Plant classified lineage-level LTR retrotransposon reference library for free-alignment methods based on Machine Learning (Version V3) [Data set]. Zenodo. http://doi.org/10.5281/zenodo.4386316. Negative instances datasets (other genomic features) are available at Zenodo: Simon Orozco Arias, Mariana S. Candamil, Paula A. Jaimes, Johan S. Piña, Reinel Tabares-Soto, Romain Guyot, & Gustavo Isaza. (2021). Negative instances for detect LTR-Retrotransposons using Machine Learning (Version 1) [Data set]. Zenodo. DOI 10.5281/zenodo.4543904.
