# Supplementary figures and images for: K-mer-based machine learning method to classify LTR-retrotransposons in plant genomes
Source: PeerJ. 2021 May 19;9:e11456. doi: 10.7717/peerj.11456 (PMC8140598; doi:10.7717/peerj.11456)

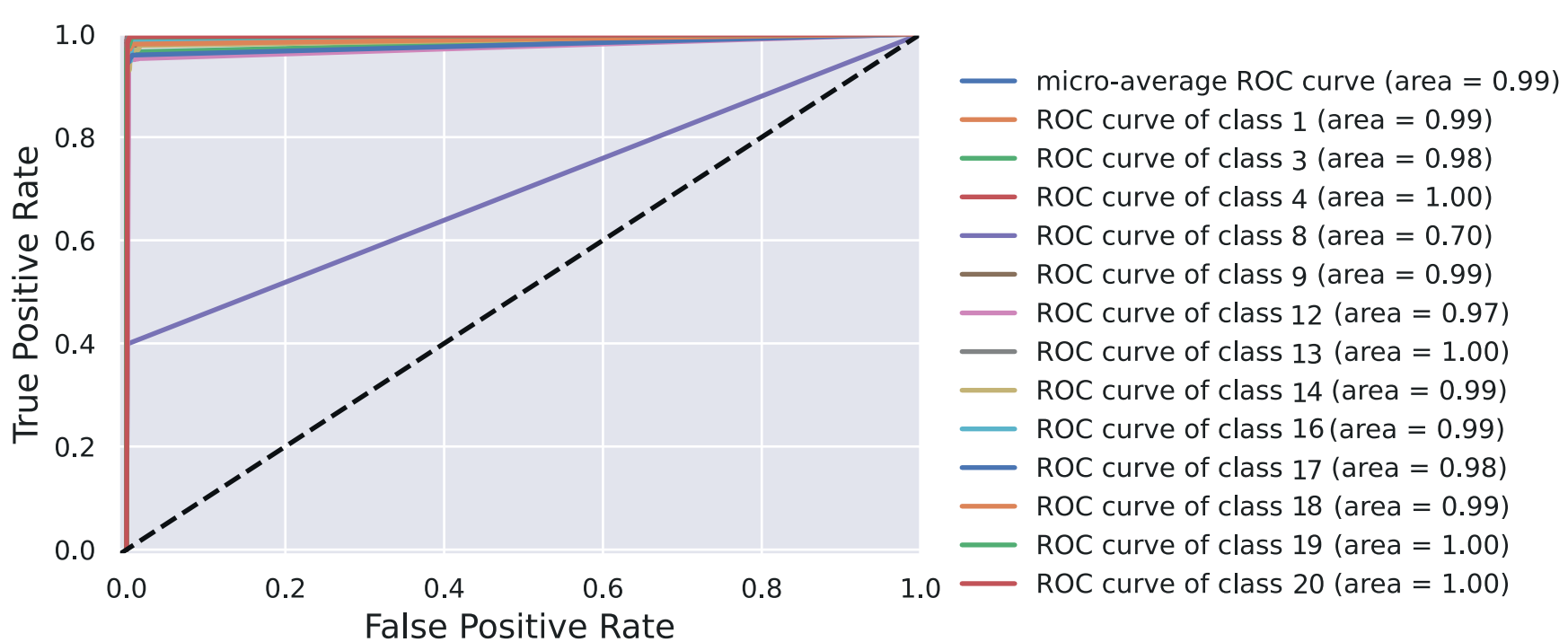

Supplement: Supplemental Information 3 — Each class (between 1 and 20) corresponds to a linage/family (See Table 4). [file peerj-09-11456-s003.pdf]

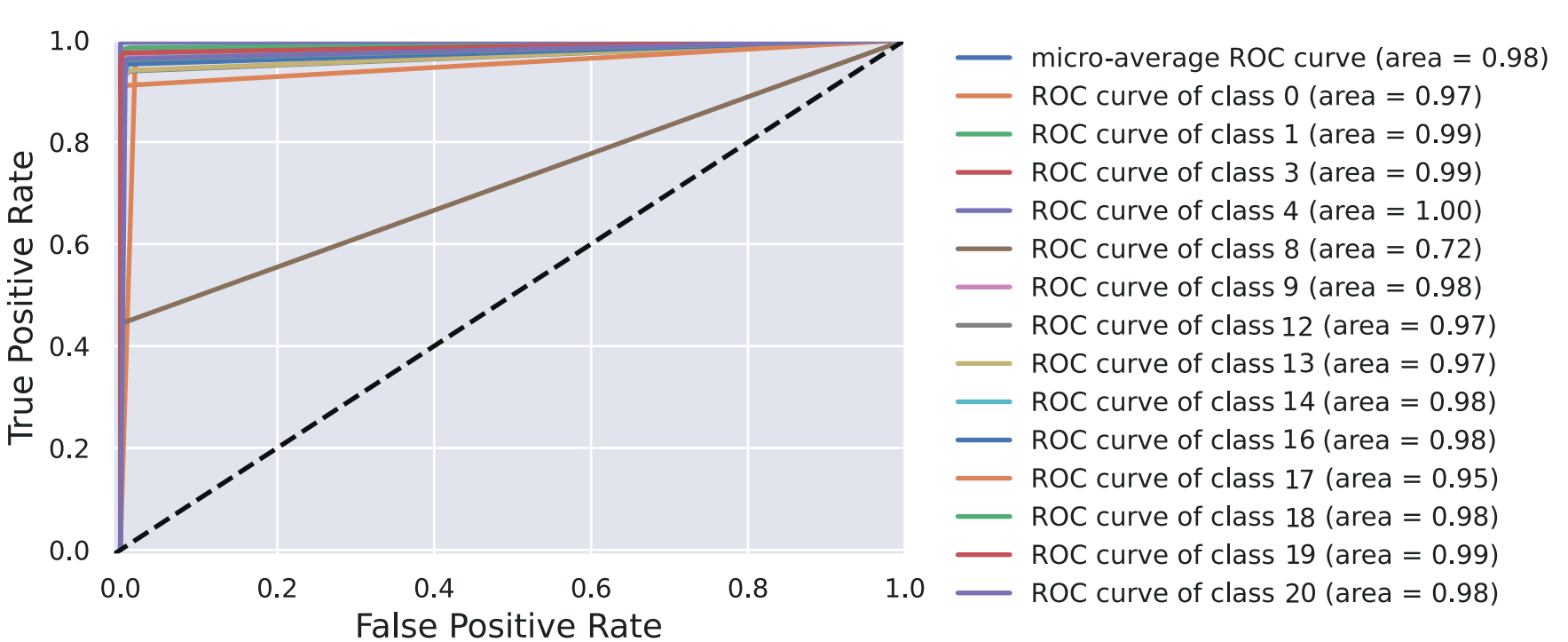

Supplement: Supplemental Information 4 — Each class (between 0 and 20) corresponds to the negative instances (for class 0) or to a linage/family otherwise (See Table 4). [file peerj-09-11456-s004.pdf]

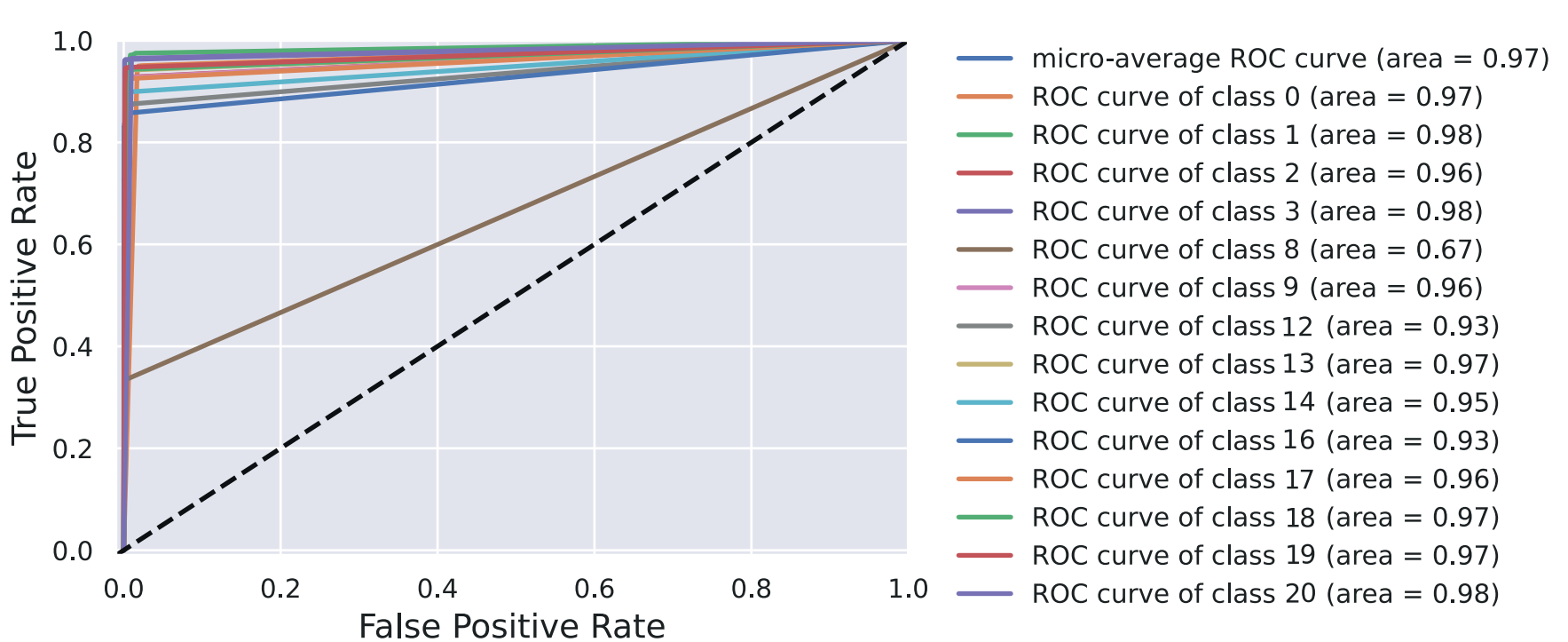

Supplement: Supplemental Information 5 — Each class (between 0 and 20) corresponds to the negative instances (for class 0) or to a linage/family otherwise (See Table 4). [file peerj-09-11456-s005.pdf]
